# Personality variation in a marine snail and heterogeneous selection in natural populations

**DOI:** 10.1093/beheco/araf146

**Published:** 2025-12-19

**Authors:** Benjamin M Nguyen, Darren W Johnson

**Affiliations:** Department of Biological Sciences, California State University, Long Beach, 1250 Bellflower Blvd, Long Beach, CA 90840, United States; Department of Biological Sciences, California State University, Long Beach, 1250 Bellflower Blvd, Long Beach, CA 90840, United States

**Keywords:** animal personality, exploration, growth, mark-recapture, multistate model, risk-taking, repeatability, survival

## Abstract

Animal personality variation is characterized by among-individual differences in behavior that are consistent across ecological contexts and over time. However, processes influencing the amount of personality variation are not well understood. In this study, we tested 1 hypothesized mechanism through which variation in personalities may be maintained: spatial variation in natural selection. Through laboratory behavioral assays, we demonstrated that 2 personality traits—exploration and risk taking—are moderately repeatable for wavy turban snails, *Megastraea undosa* (mean repeatability values = 0.320 and 0.297, respectively). We also found that there could be up to a 1.7-fold difference in among-individual variation in behavior for different populations. We next measured natural selection on these behavioral traits by experimentally transporting assayed snails to field populations in a mark-recapture study to examine the relationships between behavioral traits and growth and survival. We studied 4 populations: 2 that had an abundance of slow-moving predators (whelks, sea stars) and 2 where slow-moving predators were absent and the major predators were fast-moving species (lobsters). Selection on behavioral traits varied significantly among local populations. Depending on location, patterns of selection could be predominantly stabilizing, disruptive, or correlational. Fitness surfaces were not necessarily similar for local populations with similar predator communities, and nearby locations could have strikingly different patterns of selection. Behavioral tendencies that were associated with high fitness in 1 population could be neutral or associated with low fitness in a nearby population. Such effects likely contribute to maintaining variation in animal personality within the broader population.

## Teaser

Animals exhibit ‘personality’ variation – differences in individual exploration, boldness, etc. – but the forces promoting such variation are not always clear. In a study of wavy turban snails, we show that behavioral traits are subject to natural selection, but the pattern varies among populations. For instance, exploratory individuals exhibited high fitness in some environments, but relatively low fitness in others. Variation in selection at different locations may help maintain personality variation within the population at large.

## Introduction

Within animal populations, it is common to observe that individuals exhibit consistent differences in behavior (see reviews by Bell et al. 2009; Neimela and Dingemanse 2018; Harrison et al. 2022; Laskowski et al. 2022). For instance, measures of behaviors such as exploration, aggression, and activity are often consistent for individuals at different times and in different contexts, but variable among individuals (Gosling 2001; Sih et al. 2004; Réale et al. 2007, 2010). Such variation is thought to reflect underlying differences in a suite of characteristics often referred to collectively as temperament or personality (Dall et al. 2004; Réale et al. 2007). Some of the more commonly studied aspects of animal personalities include traits related to boldness, exploration, aggression, activity, and sociability (Gosling 2001; Réale et al. 2007; Wolf and Weissing 2012; Kaiser and Müller 2021). Personality variation is now well documented for a wide variety of taxa (reviews by Bell et al. 2009; Kralj-Fišer and Schuett 2014; Harrison et al. 2022), but the existence of consistent individual differences in behavior is still something of an evolutionary puzzle (Stamps 1991; Dall et al. 2004). Early expectations were that individual behavioral responses should be highly flexible in order to respond optimally to most situations (reviews by Lima and Dill 1990; Blumstein and Bouskila 1996). However, despite much flexibility in behavior, an appreciable component of consistent individual variation within animal populations appears to be the rule and not the exception. Such patterns call for further study of the evolutionary forces that shape personality variation (Dingemanse and Wolf 2010; Kight et al. 2013; Laskowski et al. 2022).

Theoretical models suggest that consistent individual behavioral variation can arise because of life-history tradeoffs in which behavioral traits become correlated with life-history strategies (Wolf et al. 2007). For example, bolder individuals may take more risks in the service of quicker resource acquisition and earlier reproduction whereas shyer individuals may take fewer risks but survive longer and reproduce over longer periods of time (Stamps 2007; Biro and Stamps 2008; Réale et al. 2010 ). If the fitness of these strategies is similar, the consistent individual differences that reflect personality variation may be evolutionarily stable within the population (Laskowski et al. 2022). These ideas are encapsulated in the pace of life syndrome (POLS) hypothesis, a growing body of work that describes how consistent behavioral differences can coevolve with variation in life history strategy (eg, Dingemanse and Wolf 2010; Réale et al. 2010; Dammhahn et al. 2018; Mathot and Frankenhuis 2018). Such conceptual models invoking tradeoffs in fitness components offer valuable guidance for understanding behavioral variation but still require more testing in natural settings and under different ecological circumstances.

Another mechanism that may promote consistent individual differences in behavior is variation in natural selection that may occur over space and time. In such cases, there may be a single, optimal behavioral strategy within a particular environment, but the optimal strategy within another environment may be different. For example, a bolder behavioral strategy may be optimal in low-predation environments whereas a shy strategy may be better in high-predation environments (eg, Stamps 2007; Réale et al. 2010). Variation in personality may thus be a product of environmentally driven differences in natural selection on behavioral traits, and consistent individual variation in behavior may be aligned on an axis of environmental variation. This is in slight contrast to the POLS hypothesis, which envisions individual behavioral variation to be aligned with a continuum of life history strategies with similar fitness levels (eg, bold individuals that take more risks and reproduce earlier on average may have similar expected fitness as shy individuals that take fewer risks and reproduce later). However, environmental variation may alter both the direct patterns of selection on behavioral traits and the intensity of life history tradeoffs, so the 2 mechanisms promoting behavioral variation are unlikely to be completely exclusive. Because of this link, the broader context of life history theory should be a useful guide when designing and interpreting studies of selection on behavioral traits in natural populations. For instance, examining how traits such as boldness relate to survival and growth in both high- and low-predator environments may illuminate fitness tradeoffs because relationships between boldness and mortality risk are expected to intensify with predator encounter rate (eg, Luttbeg and Schmitz 2000).

Understanding the details of selection on behavior in natural environments will be critical for understanding how variation in animal personality is maintained. Even though behavioral traits may be linked to fitness and are not as flexible as once thought, it is important to consider that some forms of selection may reduce variation. In general, it is likely that some amount of the consistent individual behavioral variation results from underlying genetic variation (Van Oers et al. 2005; van Oers and Mueller 2010; Dochtermann et al. 2015). If there is stabilizing selection, then all else equal, selection should over time erode variation in behavioral traits. Alleles associated with the optimal behavioral tendency should eventually become fixed, and as genetic variation declines in the long-term (Kimura 1965) there should be lower capacity for consistent differences in behavior among individuals. This evolutionary principle may help explain differences in the level of personality variation within populations, but also needs further testing in the field with an emphasis on quantifying both the degree of among-individual variation in the behavioral traits that reflect personality variation, and the patterns of natural selection on those behavioral traits.

Natural selection can be described by measuring the relationships between phenotype values (including behavioral traits) and the relative fitness of individuals in their environment (eg, Lande and Arnold 1983; Brodie et al. 1995; Schluter 2000; Morrissey and Sackrejda 2013). Fitness is the result of several demographic components, including growth, survival, and reproduction. And although comprehensive measures of fitness are prohibitively difficult for many species (eg, if generation times are long, individuals are difficult to track, or reproductive events are difficult to observe), selection can still be usefully measured by examining the major components of fitness that are likely to covary with the phenotypic traits of interest. This is especially true if other components of fitness are neutral with respect to the traits under consideration (Lande and Arnold 1983). In the context of personality variation, it may be particularly important to test whether behavioral traits have differing associations with different components of fitness (eg, does boldness increase growth but reduce survival?). Such tradeoffs are hypothesized to be central to the maintenance of personality variation (Stamps 2007; Réale et al. 2010) and thus warrant more testing in natural environments.

There were 2 parts to this study. First, we used repeated laboratory assays to quantify the degree to which individuals of a marine gastropod (the wavy turban snail, *Megastraea undosa*) exhibited consistent differences in measures of exploration and risk-taking—2 aspects of personality that are thought to influence fitness. We compared variance components and repeatability values for 2 regional populations and examined correlations between behavioral traits. In the second part of the study, we transported assayed snails to the field and conducted mark-recapture analyses to quantify natural selection on exploration and risk-taking tendencies within 4 field populations. Our approach to analyzing selection was sequential. We first measured the associations between behavioral traits of individuals and individual rates of growth. We next measured the association between behavioral traits and an individual's probability of survival during the duration of the study (∼180 days on average). We then examined how behavioral traits were related to expected biomass production, which was calculated from the combination of growth and survival. A major goal of the study was to simply characterize and compare patterns of selection on behaviors in 4 field populations. In addition, we tested 3 hypotheses related to the maintenance of variation in animal personality. We tested whether the patterns of selection on behavioral traits differed significantly between populations and whether any differences in selection corresponded with differences in predator community. We expected that associations between exploration and survival would be negative in environments with abundant, slow-moving predators and neutral in environments with scarcer, fast-moving predators. We also tested whether selection on exploration via growth was generally counter to selection via survival as predicted by theory. Lastly, we examined whether the degree of among-individual variation in behavioral traits was correlated with the strength of nonlinear selection on those behaviors within the local population. This last comparison was somewhat qualitative given the limited number of traits and field populations that could be studied, but in general we expected that stronger, stabilizing selection would be associated with less variation among individuals and that stronger correlational selection would be associated with stronger covariation among behavioral traits.

## Methods

### Study system

The wavy turban snail is found from Central to Baja California and inhabits rocky substrates from the intertidal zone to about 80 m deep. Previous mark recapture studies suggest these snails tend to stay on or near their home reef (Martone and Micheli 2012; McCann and Johnson 2021), making them a suitable species for this study. These snails are broadcast spawners and reach sexual maturity at around 5–7 years (∼50–70 mm shell diameter). Wavy turban snails are eaten by several different predators including whelks (*Kelletia kelletii*), sea stars (especially *Pisaster giganteus* and *Orthasterias koehleri*), lobsters *(Panulirus interruptus*), and octopus (*Octopus bimaculoides*; Schmitt 1982; Alfaro and Carpenter 1999). Juvenile and small adult snails (20–80 mm) were targeted for this study. Juvenile snails generally have greater energetic scope for variation in growth (eg, Stella and Johnson 2025), and we reasoned that if there is selection on behavioral traits via phenotype-associated differences in growth and survival, it would likely be more pronounced in smaller Wavy Turban snails as they show greater variation in growth rate (Cupul-Magaña and Torres-Moye 1996), are more susceptible to predation than larger ones (Schmitt 1981; Alfaro and Carpenter 1999), and exhibit greater variation in survival in the field (McCann and Johnson 2021).

### Collection and tagging

**Figure 1:**
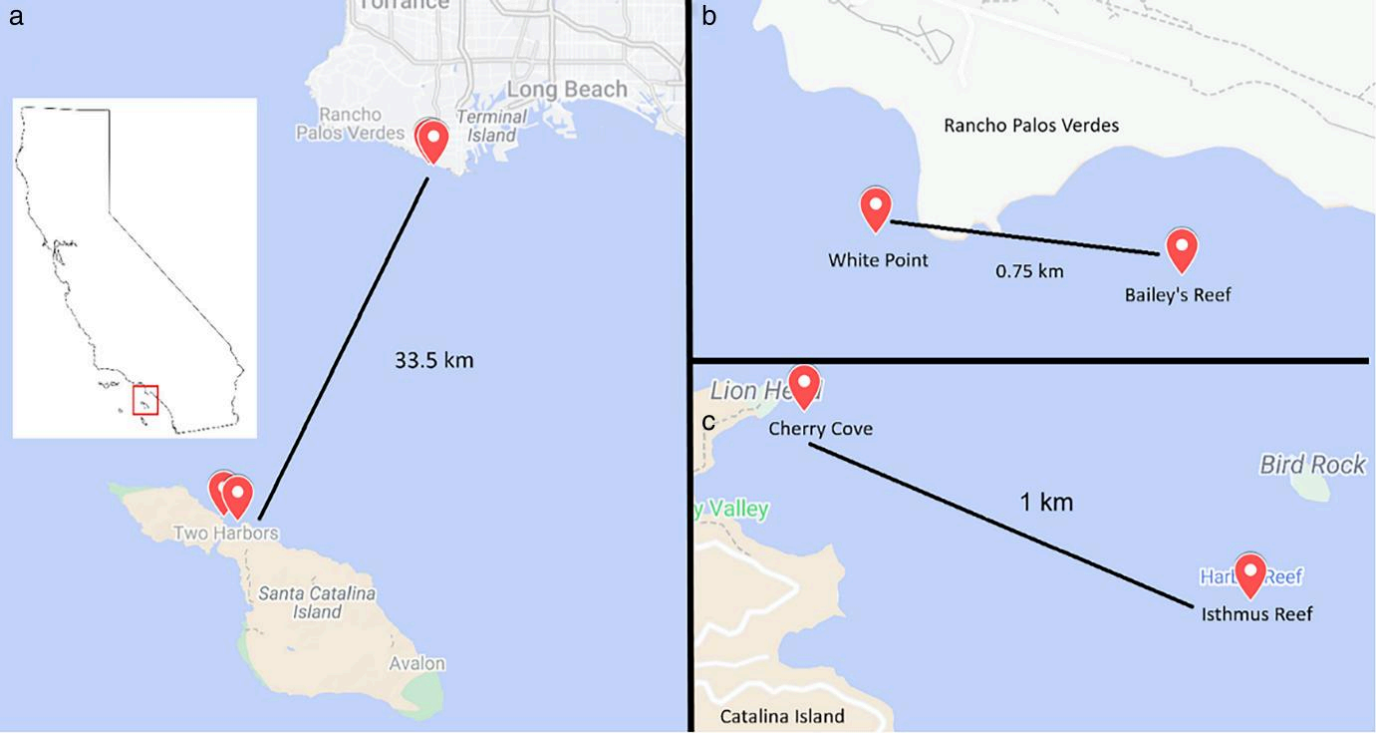
Locations of our mark-recapture studies. a) Overview of study region within Southern California, USA. b) Closeup of mainland locations (Palos Verdes Peninsula). c) Closeup of Santa Catalina Island locations. Source: Google Maps ( www.maps.google.com; accessed September 2025)

Wavy turban snails were collected on SCUBA in cohorts of 60–120 individuals from locations in our study region (Fig. 1). A total of 785 individuals were collected from the mainland region (Palos Verdes Peninsula) and 527 from the region of Santa Catalina Island. Collections were made within 500 m of our mark-recapture locations. The mainland region is characterized by a high density of predatory whelks that are relatively sedentary whereas the island region has no whelks and the main predators are spiny lobsters which are relatively scarce but fast-moving (see Appendix S1 for details and data summaries). Wavy turban snails have larvae that are planktonic for several days and locations within each region likely experience substantial exchange of larvae whereas larval exchange between island and mainland regions is likely minimal (del Próo et al. 2003). Snails were transported back to the laboratory and housed at a density of approximately 0.3 snails/L in 124-L tanks within a larger, 68,000-L recirculating system of seawater. Snails were fed a diet of *Macrocystis pyrifera* every other day but were starved for 24 h before undergoing a behavioral assay. Shortly after the snails were collected and brought back to the lab, each snail received 2 tags consisting of small discs (5 mm diameter) made using underwater paper (10 mil thickness) and printed with a numeric code. Tags were glued on opposite sides of the shell using cyanoacrylate glue and covered with nail polish topcoat to prevent scratching and deter fouling by encrusting organisms. After snails were tagged, we measured their basal shell diameter to the nearest 0.1 mm using dial calipers and measured their mass to the nearest gram using a digital hanging scale.

### Behavioral assays

After snails were collected, they were given a week to acclimate to laboratory conditions before starting repeated behavioral assays for exploration and boldness. Each assay was repeated 3 times and conducted under standard laboratory conditions. There were minor variations in water temperature (up to 2–3 °C as water in the assay tanks warmed during the course of trials) so temperature was included as a covariate in all analyses. We note that order in which snails were assayed was randomized each time. Water could warm during a single round of assays, but average water temperatures were similar across the repeat sets of trials that were separated by 5 days on average.

To measure exploration, we quantified an individual's tendency to explore a novel environment. Each snail was placed in a circular arena (28 cm diameter by 13 cm high) filled with 2 L of freshly collected seawater. Eight of these arenas were placed on a table, with a digital camera mounted on a wooden frame 1.3 m above. During the 10-min assay, snails were photographed every 10 s, for a total of 60 time-lapse photos. To determine the total path length traveled for each snail, we manually superimposed a line connecting the locations of the snails in each photo and used ImageJ to calculate the length of the path in pixels (similar to Jellison et al. 2016). To convert units of pixels to centimeters, another line was drawn across each of the 28 cm diameter arenas. We defined each snail's exploratory score as how many centimeters it moved over the duration of the assay.

To measure risk taking, we quantified an individual's response to a simulated physical threat. A snail was picked up and turned upside down by an observer. Using a blunt probe, the observer gently contacted the foot of the snail and repeated the motion until the operculum closed. The same probe was used for each trial and care was taken to keep the intensity of the probe stimulus the same across all trials. Once the operculum closed, the snail was then placed upright in a circular tank filled with seawater. Time was recorded as the time it took for the first tentacle to emerge from underneath the shell (Dahirel et al. 2021). Trials were capped at 3 min. For this assay, earlier emergence time is associated with greater risk but yields a low value. To make it so that higher values denote higher risk taking, we used the negative of the (ln-transformed) emergence time as our measure of risk taking (see Brown and Braithwaite 2004 for a similar approach). We did not identify sex of individual snails because reliably identifying the sex would require destructive sampling, and after behavioral assays snails were transported to the field for studies of growth and survival.

### Estimating repeatability of behavioral traits

All data analyses in this study were conducted using R Statistical Software (v4.2.3; R Core Team 2024). To measure the repeatabilities of exploration and risk-taking, and the correlation between these traits, we used a multivariate mixed-effects model in which the behavioral measures were analyzed together as the response variables. Shell diameter, water temperature, and assay number were included as fixed effects to account for any systematic influence on behavioral measurements. For example, even though larger snails simply move faster (Schmitt 1981) and may be expected to travel farther distances in a 10-min bout of exploration, we were interested in quantifying behavioral traits after accounting for body size. Movement speed may also vary systematically with temperature. Assay number (1, 2, or 3) was included as a fixed effect to see if snails were adjusting to the behavioral assays over time through a habituation effect (Turner et al. 2006). Snail ID number was included as a random effect to estimate among-individual (co)variance in behavioral traits. Analyses used the mcmcglmm package (Hadfield 2010) and followed the procedure outlined by Houslay and Wilson (2017). The glmm ran for 402,000 iterations with a burn-in interval of 2,000 and a thinning period of 100 iterations. Convergence for each model was assessed by verifying that autocorrelation values for MCMC-sampled parameters were approximately zero, that the posterior distributions were smooth and unimodal, and that all estimates passed the diagnostic tests of Heidelberger and Welch (1983). We used weakly informative priors based on recommendations by Houslay and Wilson (2017) and we also verified that even highly concentrated prior distributions had negligible effects on our results, likely because of the large sample sizes. For each of the MCMC samples of the posterior distribution, we calculated repeatabilities for each behavior as the among-individual component of variance divided by the total variance (ie, the sum of the among, and within-individual components). The correlation among behavioral traits was calculated as the among-individual covariance divided by the product of the square roots of the among-individual variances. We then summarized across all of the MCMC samples and used the 2.5th and 97.5th percentiles to define 95% Credible Intervals for both repeatability values and correlations among behavioral traits. For the subsequent analysis of selection, we used the Best Linear Unbiased Prediction from the mcmcglmm model to characterize each individual's behavioral traits. Importantly, we also used the uncertainty of these values to correct for known biases in selection estimates (Dingemanse et al. 2021; see details regarding growth and survival analyses below).

### Field mark-recapture study

Once a cohort of snails was assayed (70 to 100 individuals), the snails were released to 1 of 4 underwater reefs in Southern California, USA. Snails collected from the mainland were released to a mainland location, whereas snails collected from the island were released to an island location. The 2 mainland sites were located along the Palos Verdes Peninsula and included White Point and Bailey's Reef (Fig. 1). The 2 island sites were located near Santa Catalina Island and included Cherry Cove and Isthmus Reef. To quantify existing densities of snails and their predators at these locations, we counted organisms on 6, 30 × 2 m belt transects at each location. Predators included whelks, sea stars, octopus, and lobster. Cohorts of tagged snails were released monthly to a site, followed by 7 monthly revisits to survey individual growth and survival. Recapture surveys consisted of 2–3 divers searching opportunistically for tagged snails over the duration of 50 min. Once the search concluded, divers returned to the boat, where they recorded the ID of the snails found and measured individuals for shell diameter and mass. For each snail, we also recorded the number of tags present (1 or 2), as well as the state of the tags. We defined tag state as the proportion of algal growth on the tag, measured as either 0, ⅛, ¼, ⅜, ½, ⅝, ¾, ⅞, or 1 (0 = clear, 1 = completely overgrown). After snails were identified and measured, they were returned to the site of collection by divers. Details regarding our analyses of tag loss and overgrowth are described in Appendix S2.

### Behavioral traits and growth

Individual growth was measured as the change in basal shell diameter from 1 observation to the next. Like many organisms, growth in wavy turban snails follows a von Bertalanffy growth curve and growth in length tends to decrease linearly as snails get larger in size (Cupul-Magaña and Torres-Moye 1996; Martone and Micheli 2012; McCann and Johnson 2021). Since we were interested in the effects of behavioral traits independent of body size, we measured relative growth as the residuals of a linear model of growth versus initial body size. We used linear regression to estimate the relationships between relative growth and behavioral traits. Behavioral trait values were obtained from the BLUPs for each snail in the repeatability analysis and thus expressed individual behavioral tendencies after accounting for size, water temperature, and trial number. BLUPs were centered to zero, and for our analyses, were standardized by dividing by the among-individual standard deviation in values. Explanatory variables in the regression included measures of exploration, risk-taking, as well as their squared terms and product. A separate model for growth was fitted for each site, as we expected patterns to differ and because our ultimate goal was to combine functions relating behavioral traits to both growth and survival and thus biomass.

When the explanatory variables in a regression analysis have imperfect repeatability, the estimated regression coefficients will be biased (this is the so-called error-in-variables problem; Weisberg 2014). Dingemanse et al. (2021) show how such biases can be corrected in the context of selection analyses, and we followed their guidelines in our analyses. For each of the MCMC samples of the posterior distributions in our repeatability analysis, we calculated the expected attenuation bias for each of the linear, quadratic, and correlational terms (see formulas in the supporting information of Dingemanse et al. 2021). Posterior distributions were approximately normal and expected biases were summarized by the mean and standard deviations. For the analysis of growth, each of the estimated regression coefficients were corrected for bias by dividing by the mean attenuation bias derived from the repeatability analysis. Confidence intervals for the bias-corrected regression coefficients were calculated using a simulation procedure to incorporate uncertainty in the bias corrections. For each of 1,000 iterations, a set of regression coefficients was drawn from a multivariate normal distribution with the means and (co)variances taken from the parameter estimates of the regression model. A set of bias corrections was also drawn from a multivariate normal with the posterior means and (co)variance matrix derived from the MCMC samples of the attenuation biases calculated from the repeatability analysis. For each iteration, the simulated regression coefficients were divided by the simulated bias corrections. The 2.5th and 97.5th percentiles of these combined distributions were used to generate confidence intervals that included the uncertainty in bias corrections. Uncorrected parameters relating behaviors to growth and survival are included in Appendix S3.

### Behavioral traits and survival

Mark-recapture analysis was used to estimate survival and recapture probabilities of snails at the different sites. Using the data from monthly recapture surveys at each site, we formed an encounter history for each snail—a summary of whether the individual was recaptured or not recaptured on each survey. We then used the *RMark* package in R (Laake 2013) which uses the *Mark* software (White and Burnham 1999) to analyze all the encounter histories for each site. The essence of this analysis is to find the values of survival and recapture probabilities that were most likely to have produced the observed encounter histories. Because tag loss and tag overgrowth were substantial, we used a multistate mark recapture model to represent the various possible states of the tags. For the different states, our model included 2 observable states: 2 (2 tags present), and 1 (1 tag present), and 2 unobservable states: L (both tags fallen off) and G (both tags overgrown). The multistate mark-recapture model has 3 processes to be estimated: survival (*S*), recapture (*r*), and state transition (Ψ) probabilities. In this study, survival was expressed as a function of behavior and transition probabilities were based on our analysis of tag loss and tag overgrowth (see above). By accounting for these unobservable states, we were able to adjust our recapture probabilities by accounting for individuals that were still alive but not recapturable and thus obtain more accurate estimates of survival.

In the model, snails would start in the 2-tag state and either remains in that state or transition to any of the other states as time progressed. Since states 2 and 1 were both observable states, we let the model estimate the transition probability from state 2 to state 1 using the data. For the unobservable states, transition probabilities to these states were calculated from our separate analyses of tag loss and overgrowth (Appendix S2). Transition probabilities describing the gain of a tag or the undoing of overgrowth were fixed at zero.

To evaluate the effects of behavioral traits on survival, we modeled survival as a function of behavioral trait values (standardized BLUPs from the repeatability analysis) by adding them in as individual covariates to the function describing survival (S). Within the mark-recapture model, our component model for survival was 1 in which the logit of survival was described as a linear model of the following explanatory variables: Diameter, Diameter^2^, Exploration, Risk taking, Exploration^2^, Risk taking^2^ and Exploration by Risk taking (see Gimenez et al. 2009; Waller and Svensson 2016 for similar approaches). Coefficients describing the relationship between behavioral traits and survival were used to describe the selection surface. Shell diameter was included as an individual covariate, as previous research has shown that survival in wavy turban snails can be size-dependent, and sometimes in a nonlinear fashion (McCann and Johnson 2021; Tuskes 2021; see also Alfaro and Carpenter 1999). Recapture probability was allowed to vary during each census because although the search procedure was the same, there were slight variations in water visibility and search effort that may have affected recapture probability. Transition probabilities (Ψ) describing tag loss were estimated from the encounter history or were fixed as described above. We used the same bias-correction procedure as in the growth analysis, except that the linear model coefficients and expected attenuation biases were first calculated for the logit of survival and combined on that scale before being back-calculated to express expected survival (and appropriate confidence interval end points) on a scale bounded by 0 and 1.

### Behavioral traits and biomass production

For wavy turban snails, biomass production is likely to be a very good proxy for individual fitness because reproductive output is highly correlated with body mass (Martone and Micheli 2012). Measuring biomass was also more practical because measuring reproductive output directly would require destructive sampling and would have added a significant workload to an already labor-intensive study. To quantify biomass production, we combined the functions relating behavioral traits to both survival and growth to describe the relationship between behaviors and relative biomass production. To do this, the function describing daily growth in diameter as it relates to behaviors was applied to a starting diameter of 45 mm (the average size of snails entering our study) to project size at each day. The projected size increments were then used to calculate expected survival, which was dependent on both behavior and size (see previous section for details). In this way we captured both the direct effects of behavior on survival, as well as any indirect effects that arise via relationships between behavior and growth in combination with size-dependent survival. We took the product of daily survival probabilities across 180 days to calculate expected survival. A period of 180 days was chosen because it is a round number near the average duration of our field studies. Expected survival was then multiplied by expected mass per individual to get net biomass production. Tissue mass scales isometrically with diameter (McCann and Johnson 2021), so diameter was cubed to convert length to relative mass. To express relative fitness, biomass production associated with each combination of exploration and risk-taking exhibited by individuals in our sample was divided by the expected biomass production, averaged across all individuals. In this way, average, relative fitness was set to 1 within each population. To test for differences in the biomass fitness surfaces among study locations, we used a randomization test that incorporated uncertainty in both growth and survival functions (see Appendix S2). Uncertainty affects both Type I and Type II error, and to offset these effects, we consider *P*-values < 0.10 as support worth considering. We note that for a finite amount of effort, there is a tradeoff between measuring selection in several populations vs obtaining high precision estimates of selection coefficients in 1 or 2 populations. Rather than conducting hypothesis tests on each parameter of all components of the fitness functions, our approach was to report parameters and their accompanying uncertainty, but to limit the focus of hypothesis testing to the comparison of patterns of selection via differential biomass at the different locations.

### Selection gradients

We quantified selection by evaluating the relationships between behavioral traits and relative biomass production. Because the functions describing relative biomass were nonlinear and somewhat complex, we calculated selection gradients by empirically evaluating the partial derivatives of the fitness surface (W_z_) at the observed combinations of phenotype values. In particular, linear selection gradients (β values) were calculated as:


$$\beta ={\bar{W}}^{-1}N^{-1}\overset{N}{\underset{i=1}{\sum }}\frac{\partial W_{z}}{\partial z}{|}_{z=z_{i}}$$

where *z* is the focal phenotype, *N* is sample size and, $\bar{W}$ is mean fitness (Janzen and Stern 1998; Morrissey and Goudie 2022). Nonlinear gradients (γ values) were calculated as:


$$\gamma ={\bar{W}}^{-1}N^{-1}\overset{N}{\underset{i=1}{\sum }}\frac{\partial^{2}W_{z}}{\partial z\partial z^{\prime }}{|}_{z=z_{i}}$$

Such an approach to calculating selection coefficients is preferred when the fitness surface departs from a quadratic function (Phillips and Arnold 1989; Walsh and Lynch 2018), and several of the fitness functions in this study were substantially more complex than a quadratic function. In our calculations, partial derivatives were calculated numerically using the *numDeriv* package in R (Gilbert and Varadhan 2019). Selection gradients were calculated for each of the 4 study locations. In addition, we calculated a set of selection gradients for the mainland region and a set of selection gradients for the island region. This was accomplished by applying the expressions above to the combined biomass functions for the 2 study locations within each region (ie, $W_{z}=0.5W_{1_{z}}+0.5W_{2_{z}}$, where *W*_1_ and *W*_2_ are the fitness functions for location 1 and location 2). We reasoned that because gene flow within these regions is likely to be high, but gene flow between regions is likely to be low, the combined fitness function would be more representative of selection within the region and therefore more relevant to compare with measures of repeatability.

## Results

### Behavioral assays

For the exploration assay, the average snail traveled 30.5 cm in 10 min (SD = 30.26, Range = 0–203.1 cm). Despite testing a range of snails with varying shell diameters (Mean = 44.6 cm, SD = 9.86, Range = 16.7–79.2), there was no significant effect of body size on movement in these tests (Table 1). However, snails tended to move more with increasing water temperature. There was moderate variation in water temperature during behavioral assays (Mean = 18.77 °C, SD = 1.4, Range = 15.6–22.9), and a 1 °C increase in temperature would on average increase distance traveled by a factor of e^0.066^ (or a 6.8% increase; Table 1). The significant effect of assay number suggests a habituation effect in which snails moved greater distances in later assays (Table 1). Individuals traveled an average of 27.16 cm during the first assay compared with 33.96 cm during the third assay—a 25% increase in distance traveled. These results suggest a moderate increase in exploration as snails became habituated to laboratory conditions. In addition to these results, there was strong evidence of consistent; among-individual differences in exploration behavior (see random effects in Table 1). When expressed as a fraction of the total variation (among-individual plus residual), repeatability of exploration behavior was estimated to be 0.320 (95% Credible Interval: 0.274, 0.361; Table 1).

**Table 1:** Summary of the generalized linear mixed-effects model used to analyze behaviors.

*Fixed effects*						
Component of response	Explanatory variable	Mean	l-95% CI	u-95% CI	Eff.samp	pMCMC
Exploration	Intercept	2.912	2.858	2.964	4000.0	<2.5 × 10^−4^
Risk taking	Intercept	−3.171	−3.201	−3.140	3821.9	<2.5 × 10^−4^
Exploration	Trial number	0.129	0.079	0.174	4000.0	<2.5 × 10^−4^
Risk taking	Trial number	0.062	0.036	0.089	4000.0	<2.5 × 10^−4^
Exploration	Diameter	−0.002	−0.008	0.003	4000.0	0.404
Risk taking	Diameter	−0.021	−0.024	−0.018	4000.0	<2.5 × 10^−4^
Exploration	Water temperature	0.068	0.034	0.102	4000.0	<2.5 × 10^−4^
Risk taking	Water temperature	0.087	0.069	0.105	4113.4	<2.5 × 10^−4^

*Random effects*						
	Trait	Mean	l-95% CI	u-95% CI	Eff.samp	
*Among-individual (co)varation*	Exploration	0.436	0.362	0.508	4000	
	Covariance	0.100	0.070	0.130	4000	
	Risk taking	0.131	0.109	0.155	4000	
*Within-individual (co)varaition*	Exploration	0.923	0.862	0.983	4000	
	Covariance	0.049	0.025	0.076	4203.2	
	Risk taking	0.311	0.291	0.332	4470.7	

A total of 1312 individuals were assayed, and each individual was scored on 3 repeated assays per behavioral trait.

For the risk-taking assay, the average snail took 30.86 s to emerge from their shell after being disturbed (SD = 33.8; Range = 1–180 s). Smaller individuals took significantly less time to emerge from their shells compared with larger individuals (Table 1). Water temperature also had a significant effect on risk taking (Table 1), with a 1 °C increase resulting in an increase of emergence time by a factor of e^0.087^ (or a 9.0% increase). Assay number had a significant effect (Table 1), and snails tended to emerge faster as they went through the assay the second and third time. The repeatability of our measure of risk taking was estimated to be 0.297 (95% Credible Interval: 0.253, 0.339). After accounting for variation in behavioral scores associated with fixed effects, individual tendencies for exploration and risk taking were moderately correlated (*r* = 0.42; 95% Credible Interval: 0.309, 0.533). Examining the distributions of individual random effects revealed moderate differences in the mean exploration values for the 2 source populations [Palos Verdes (mainland) = 0.137, Catalina Island = −0.155; values in SD units], but only slight differences in mean risk-taking values (Palos Verdes = −0.0248, Catalina Island = 0.0287; values in sd units).

**Figure 2:**
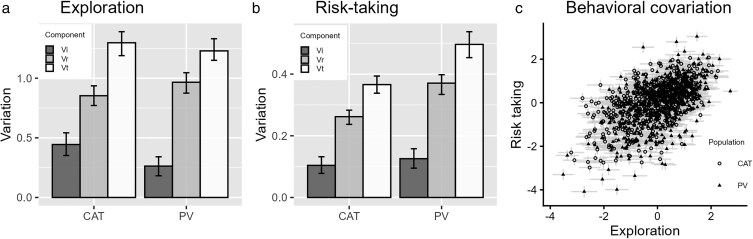
Summary of behavioral trait variation in populations of wavy turban snails. Panels a and b illustrate the consistent, among-individual variation in behaviors (Vi; dark gray bars), as well as the within-individual variation (Vr; light gray bars), and estimated total (Vt; white bars). Error bars illustrate 95% Credible Intervals. Data are shown for Santa Catalina Island (CAT) and mainland locations (PV). Panel c illustrates the correlation among behavioral traits. Dots show the values of the Best Linear Unbiased Predictions for the mean value of each individual's behavioral scores. Error bars represent ±1 SE.

Beyond these overall patterns, analyzing behavioral variation separately for the 2 source populations revealed some differences in the components of behavioral variation. For exploration, snails from Santa Catalina Island exhibited greater among-individual variation, and less within-individual variation than their counterparts from the mainland population (Fig. 2a). This resulted in a greater repeatability value within the Catalina Island population [0.346 95% Credible Interval: (0.289, 0.408)] compared with Palos Verdes [0.214 (0.153, 0.269)]. For risk-taking behavior, there was slightly more among-individual variation in the Palos Verdes population (Fig. 2b), but this was accompanied by greater within-individual variation, resulting in comparable values of repeatability [Palos Verdes = 0.252 (0.201, 0.313); Catalina Island = 0.281 (0.216, 0.330)]. Correlations among behavioral traits were similar for the 2 source populations [Palos Verdes = 0.370 (0.173, 0.558); Catalina Island = 0.381 (0.212, 0.526)].

### Behavioral traits and growth

**Figure 3:**
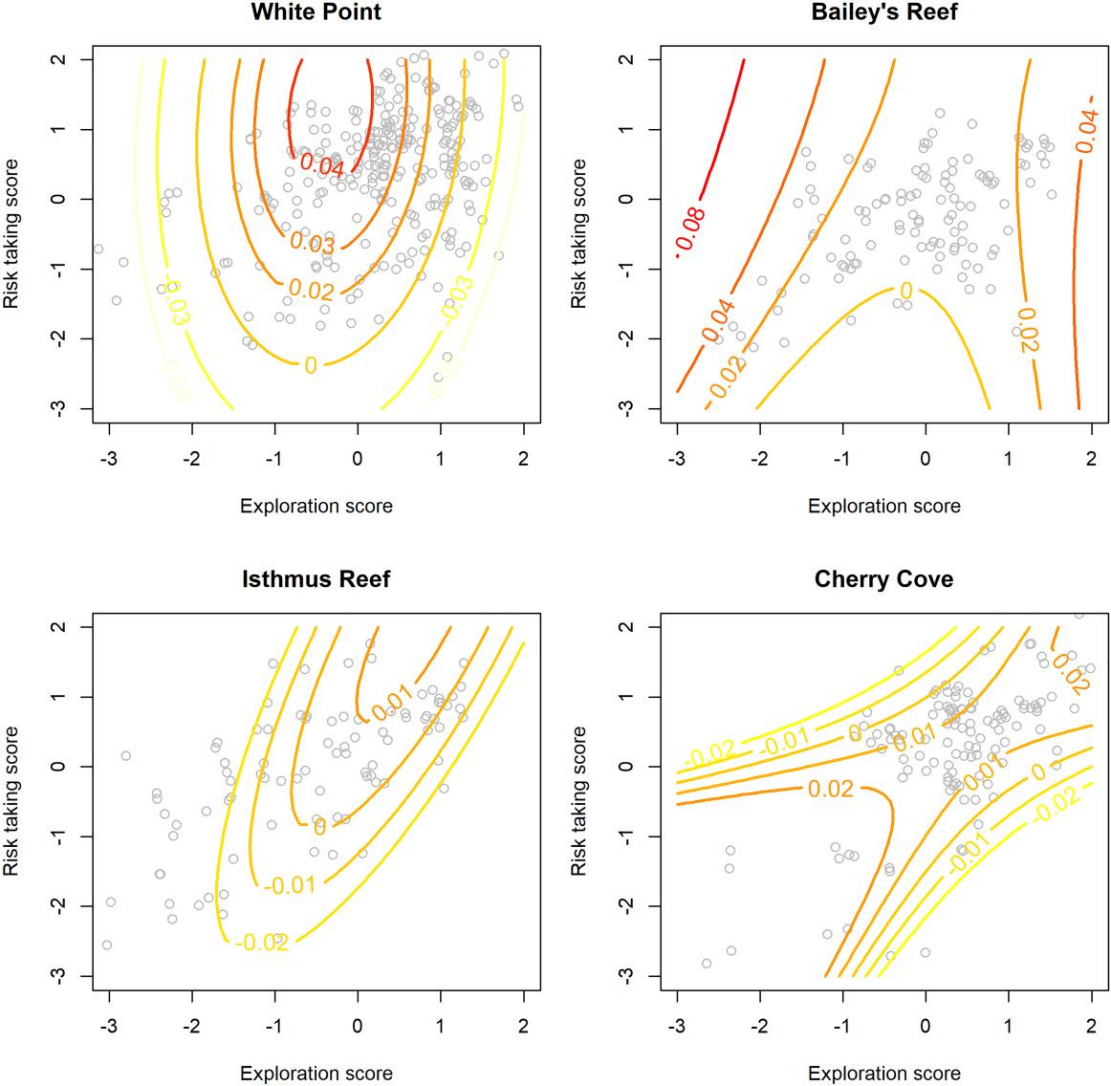
Nonlinear regression surfaces illustrating the associations between behavioral traits and growth for each of our study populations. Behavioral score values on the *x* and *y* axis is the Best Linear Unbiased Predictions from the repeatability analysis and express the mean value of individual's behavioral scores expressed as standard deviations from the mean. Contour values indicate the expected growth in shell diameter (mm per day). Red contours = high growth, orange = moderate growth, yellow = low growth. Mainland populations (Palos Verdes) are represented in the top row and island populations (Catalina) are represented in the bottom row.

Average growth rates differed appreciably among the 4 study sites. Snails grew the fastest at White Point [0.032 ± 0.002 (SE) mm/day], then Cherry Cove (0.010 ± 0.002 mm/day), Bailey's Reef (0.009 ± 0.001 mm/day), and Isthmus Reef (0.002 ± 0.002 mm/day). Relationships between behavioral traits and growth rates also differed between sites, with the most compelling statistical support for differences between Bailey's Reef and the other locations (Randomization test *P*-values were 0.03, 0.07, and 0.11 for comparisons with White Point, Bailey's Reef and Cherry Cove; other *P*-values were greater than 0.15). Associations between behavioral traits and growth can be viewed as a component of selection, and at White Point, selection on both exploration and risk taking was predominantly stabilizing, indicated by a fitness surface optimum within the range of trait values (Fig. 3), and quadratic coefficients of −0.018 for exploration and −0.003 for Risk taking (Table 2). There was also a component of directional selection as expected growth was maximal for those snails exhibiting higher-than-average risk-taking scores and lower-than-average exploration scores (Fig. 3). At Bailey's Reef, the relationship between behavioral traits and growth suggested disruptive selection on exploration but little-to-no selection on risk taking (Fig. 3; Table 2). At Isthmus Reef, there was some evidence of stabilizing selection on exploration (Table 2) and a slight tendency for snails with a combination of high exploration and high risk taking to grow slightly faster (Fig. 3), though the statistical evidence for correlational selection was not significant at the *α*=0.05 level. At Cherry Cove, evidence of selection was weaker overall (Table 2), and the shape of the fitness surface suggested some directional selection for lower exploration and stabilizing selection on risk taking.

**Table 2:** Summary of the functions relating exploration and risk taking to growth in shell diameter (mm/day).

White point	Estimate	LCL	UCL	Bailey's reef	Estimate	LCL	UCL
Intercept	0.036	0.028	0.043	Intercept	0.009	0.004	0.013
Exploration	−0.014	−0.029	−0.001	Exploration	0.001	−0.009	0.010
Risk taking	0.009	−0.002	0.021	Risk taking	0.004	−0.007	0.015
Exploration^2^	−0.018	−0.042	0.000	Exploration^2^	0.010	−0.013	0.035
Risk taking^2^	−0.003	−0.022	0.014	Risk taking^2^	−0.003	−0.019	0.013
Interaction	0.003	−0.021	0.027	Interaction	−0.003	−0.025	0.016

Isthmus reef	Estimate	LCL	UCL	Cherry cove	Estimate	LCL	UCL
Intercept	0.007	−0.002	0.015	Intercept	0.015	0.007	0.022
Exploration	−0.004	−0.021	0.015	Exploration	−0.004	−0.015	0.008
Risk taking	0.006	−0.015	0.027	Risk taking	−0.005	−0.018	0.009
Exploration^2^	−0.015	−0.034	0.005	Exploration^2^	−0.002	−0.023	0.018
Risk taking^2^	−0.006	−0.034	0.024	Risk taking^2^	−0.010	−0.029	0.012
Interaction	0.012	−0.024	0.043	Interaction	0.020	−0.009	0.052

Behavioral scores were values of the Best Linear Unbiased Predictions from the repeatability analysis. Behavioral traits were expressed as standard deviations from the mean and response is the absolute, rather than relative value of expected growth.

### Behavioral traits and survival

Mean survival probabilities differed slightly among the 4 locations. The highest monthly survival was seen at Cherry Cove (0.9916), followed by Bailey's Reef (0.9911), Isthmus Reef (0.9898), and White Point (0.9896). Mean recapture probabilities varied between the 4 locations, and recapture probabilities also varied between individual surveys at each location. Recapture probabilities were highest at White Point (0.672 ± 0.054 SE), followed by Cherry Cove (0.450 ± 0.047), Bailey's Reef (0.285 ± 0.065), and Isthmus Reef (0.187 ± 0.045).

**Figure 4:**
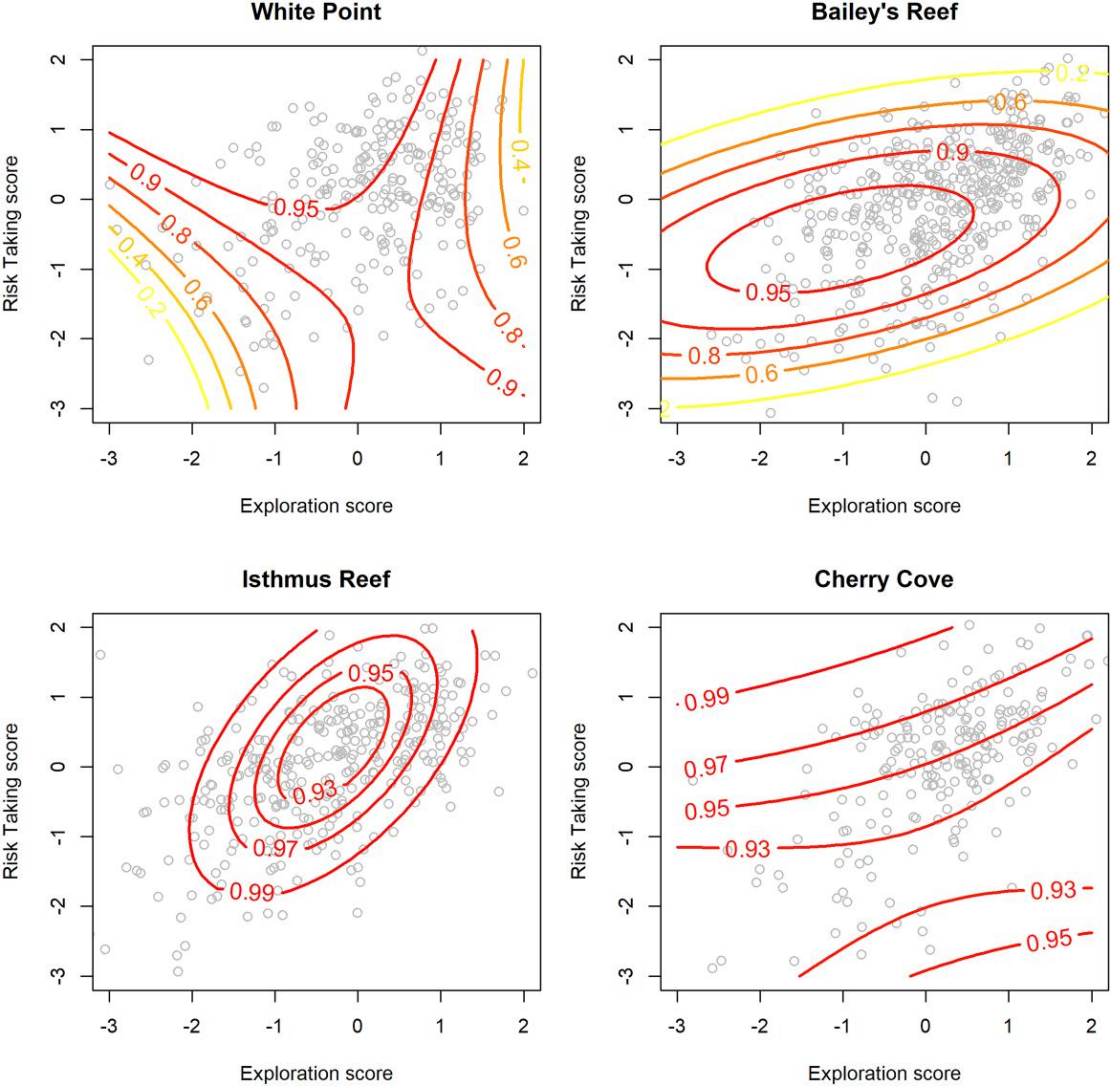
Relationships between behavioral traits and survival of wavy turban snails. Behavioral score values on the *x* and *y* axis is expressed as standard deviations from the mean, and points represent individual means (BLUPs obtained from the repeatability analyses). Contour values indicate survival probability over 6 months. Red contours = high survival, orange = moderate survival, yellow = low survival. Mainland populations (Palos Verdes) are represented in the top row and island populations (Catalina) are represented in the bottom row.

The relationships between behavioral traits and survival were different among sites (Fig. 4), and although there was some evidence that the shape of the behavior-to-survival surface could differ between nearby locations (eg, a randomization test comparing relationships for White Point and Bailey's reef yielded a *P* value of 0.095), the strongest contrast was between mainland, high-predator locations and island, low-predator locations (Fig. 4). Patterns of survival suggest stronger, and nonlinear selection on behaviors in the mainland populations. For instance, at White Point, there was a strong convex relationship between exploration and survival, suggesting a component of stabilizing selection on this trait (Fig. 4). There was only a weak association between risk taking and survival, with higher risk taking corresponding to a slight increase in survival. We remind the reader that our use of the phrase risk taking was a convenient shorthand for latency of emergence after a simulated threat in the lab. These results suggest that this trait may not necessarily reflect mortality risk incurred in the field. At Bailey's Reef, there was a component of stabilizing selection on both behaviors, indicated by the negative nonlinear coefficients (Table 3) and a localized peak in the response surface (Fig. 4). Survival declined sharply for snails that exhibited tendencies for either high risk taking or low risk taking. In a similar manner, snails with either high exploration or low exploration scores tended to have lower survival, though these declines were not as severe (Fig. 4). At the island locations, the associations between behavioral traits and survival were much weaker (see values of contour lines in Fig. 4), and the estimated coefficients were, in general, small compared with the accompanying uncertainty (Table 3). At Isthmus Reef, the surface was slightly convex, suggesting a small component of disruptive selection (Fig. 4) though the effects appear to be minor (Table 3). At Cherry Cove, there was a slight tendency for snails with either the highest or lowest risk-taking scores to exhibit higher survival, and little-to-no association between exploration and survival (Fig. 4). Estimated recapture probabilities varied among surveys, likely reflecting temporal differences in environmental conditions (especially water visibility during surveys). Accounting for such variation is necessary, but we note that versions of the models that grouped recapture probabilities into fewer levels (eg, similar probabilities during early, middle, and late phases of the study) resulted in little to no change in the coefficients relating survival to behavioral traits. We retained the full model with recapture probability estimated separately for each survey (see Appendix S3 for estimates of recapture probabilities).

**Table 3:** Summary of survival coefficients estimated by our mark-recapture analyses.

			95% CI					95% CI	
White point	Estimate	SE	LCL	UCL	Bailey's reef	Estimate	SE	LCL	UCL
Intercept	16.556	3.961	8.664	24.289	Intercept	−0.444	1.906	−4.173	3.306
Diameter	−0.403	0.143	−0.684	−0.137	Diameter	0.223	0.084	0.057	0.388
Diameter^2^	0.003	0.001	0.001	0.005	Diameter^2^	−0.002	0.001	−0.004	−0.001
Explore	−0.636	0.323	−1.267	−0.042	Explore	−0.186	0.348	−0.862	0.482
Risk taking	0.678	0.270	0.191	1.204	Risk taking	−0.597	0.363	−1.363	0.050
Explore^2^	−0.480	0.383	−1.334	0.153	Explore^2^	−0.110	0.736	−1.720	1.353
Risk taking^2^	0.195	0.416	−0.609	1.063	Risk taking^2^	−0.910	0.522	−2.038	−0.006
Explore × Risk taking	−0.507	0.505	−1.552	0.448	Explore × Risk taking	0.369	0.728	−0.953	1.899

			95% CI					95% CI	
Isthmus Reef	Estimate	SE	LCL	UCL	Cherry Cove	SE	SE	LCL	UCL
Intercept	−3.247	3.918	−10.776	4.521	Intercept	0.803	2.298	−3.856	5.386
Diameter	0.304	0.172	−0.046	0.633	Diameter	0.147	0.107	−0.067	0.363
Diameter^2^	−0.003	0.002	−0.006	0.001	Diameter^2^	−0.001	0.001	−0.004	0.001
Explore	0.932	1.250	−1.423	3.310	Explore	−0.231	0.282	−0.806	0.336
Risk taking	−0.706	1.007	−2.556	1.258	Risk taking	0.541	0.369	−0.173	1.238
Explore^2^	1.044	1.773	−2.421	4.450	Explore^2^	−0.011	0.416	−0.889	0.835
Risk taking^2^	0.589	1.090	−1.590	2.916	Risk taking^2^	0.188	0.498	−0.777	1.174
Explore × Risk taking	−0.854	1.958	−4.854	2.814	Explore × Risk taking	−0.158	0.631	−1.423	1.185

Estimated parameters are coefficients relating the logit of survival to the behavioral traits of snails in our study. Behavioral traits are Best Linear Unbiased Predictions from the repeatability analysis and the effects of uncertainty in these predictions have been corrected for in the analysis (see main text). BLUPs are expressed as standard deviations from the mean. Terms for snail diameter were included because survival depends on size and we needed to account for the fact that snails entered the study at different sizes.

### Behavioral traits and biomass production

**Figure 5:**
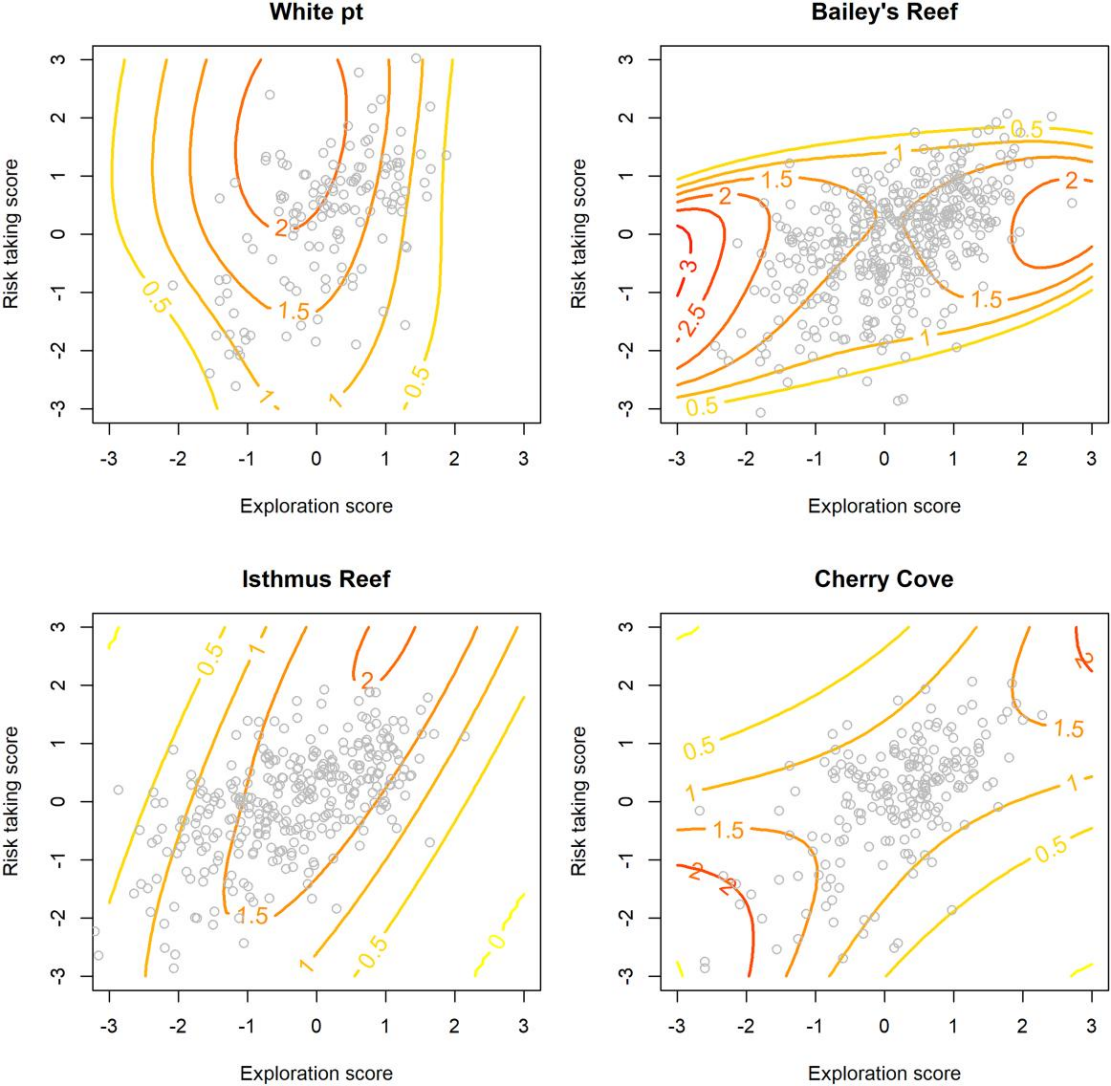
Fitness surfaces illustrating the relationships between behavioral traits and expected biomass production for our study populations. Behavioral score values on the *x* and *y* axis is expressed as standard deviations from the mean, and points represent individual means. Contour values indicate relative biomass production over 6 months, with 1 being the average biomass production of individuals at that site. Red contours = high biomass production, orange = moderate biomass production, yellow = low biomass production. Mainland populations (Palos Verdes) are represented in the top row and island populations (Catalina) are represented in the bottom row.

When we combined the functions relating behaviors to growth with functions relating behaviors to survival, it revealed that the patterns of selection through differential biomass production differed among the 4 sites (Fig. 5, Table 4). However, the differences were idiosyncratic, and nearby locations did not necessarily have similarly shaped fitness surfaces. Patterns of selection at the mainland location of White Point were significantly different from both the island location of Cherry Cove (Randomization test *P* < 0.001) and the nearby mainland location of Bailey's Reef (*P* = 0.05) but were indistinguishable from the island location of Isthmus Reef (*P* = 0.28). Comparisons among Bailey's Reef, Isthmus Reef, and Cherry Cove had marginal levels of statistical significance (Bailey's vs Isthmus *P* = 0.06; Bailey's vs Cherry *P* = 0.100; Isthmus vs Cherry *P* = 0.105) suggesting moderate differences in patterns of selection. At White Point, the fitness surface was convex, with a peak occurring at lower-than-average exploration and higher-than-average risk taking. In contrast, at the nearby location of Bailey's Reef, the fitness surface was saddle-shaped with increased biomass production associated with either high or low exploration scores, and lower biomass production associated with both high and low risk-taking scores. At Isthmus Reef, snails with higher risk-taking scores had higher biomass production and the fitness surface was convex such that increased biomass was associated with intermediate exploration scores (Fig. 5). At Cherry Cove, the fitness surface was slightly saddle-shaped with regions of high fitness corresponding to combinations of either high exploration and high risk taking, or low exploration and low risk taking. The fitness surfaces were aligned on a diagonal at both island locations (Fig. 5, bottom panels), indicating that the combinations of exploration and risk-taking values were important. In contrast, at the mainland locations, fitness was associated with behavioral trait values in a more additive manner (Fig. 5, upper panels).

**Table 4:** Summary of selection gradients estimated in this study by analyzing the relationships between behavioral traits and biomass production.

Region	Population	Summary	*β*_Exploration_	*β*_Risk-taking_	*γ*_Exploration_	*γ*_Exploration:Risk-taking_	*γ*_Risk-taking_
**Palos Verdes**	White Pt.	Mean	−0.287	0.131	−0.301	−0.038	−0.051
		SE	0.092	0.078	0.121	0.063	0.068
	Bailey's Reef	Mean	0.003	−0.011	0.202	−0.011	−0.322
		SE	0.043	0.033	0.099	0.057	0.121
	Pooled	Mean	−0.152	0.060	−0.162	−0.029	−0.157
		SE	0.044	0.032	0.086	0.047	0.082
**Santa Catalina Island**	Isthmus Reef	Mean	0.069	0.040	−0.308	0.132	−0.060
		SE	0.047	0.05	0.127	0.089	0.108
	Cherry Cove	Mean	−0.055	0.001	−0.049	0.213	−0.179
		SE	0.063	0.079	0.107	0.111	0.124
	Pooled	Mean	−0.008	−0.009	−0.191	0.171	−0.118
		SE	0.024	0.026	0.049	0.032	0.052

*β* and *γ* values represent the variance-standardized linear and nonlinear gradients of the bivariate fitness surfaces.

### Selection gradients

Selection gradients reflect the patterns of the fitness surface but also incorporate the distribution of phenotypes at each location to describe the direct patterns of selection on traits. The selection gradients estimated in this study (Table 4) indicate that behavioral traits that were selected for in 1 environment (positive *β* values) may experience little-to-no selection or may be selected against in nearby populations (negative *β* values). For instance, exploration was selected strongly against at White Point (*β* = −0.287), but at nearby Bailey's reef, there was minimal directional selection on this trait (*β* = 0.005). Similarly, the strength of nonlinear selection differed in magnitude between nearby populations and in some instances varied from concave (negative *γ* values characteristic of stabilizing selection if the fitness optimum is within the data range) to convex (positive *γ* values characteristic of disruptive selection; Table 4).

All else equal, the level of consistent, among-individual variation in behavioral traits will be higher if there is more genetic variation underlying the differences in behavior among individuals. And because the level of genetic variation can be shaped by selection, it was hypothesized that stronger stabilizing selection would be associated with lower among-individual variation. However, we observed little correspondence between selection measured at the regional scale (gradients from the pooled fitness surfaces; Table 4) and measures of consistent, among-individual (co)variation. For instance, among-individual variation in exploration was notably higher in the Catalina Island population (see Fig. 2), but this was also the region with the strongest stabilizing selection on exploration (Table 4). Similarly, the covariation in exploration and risk taking was similar both mainland and island populations (see Fig. 2, panel 3) despite correlational selection being strong and positive in only the Santa Catalina region (Table 4).

## Discussion

Snails in our study exhibited a significant amount of repeatable, among-individual variation in both exploration and risk-taking (overall repeatability values of 0.320 and 0.297, respectively). Variation in these behavioral scores is thus informative about overall patterns of variation in personality, and *average* behavioral scores (especially across 3 replicate assays) provided a reliable measure of the consistent differences in personality that exist among individuals and may be important for their growth and survival in the field. Repeatabilities of exploration and risk taking of wavy turban snails are similar in value to repeatabilities of exploration and antipredator behaviors observed in many other species including birds (eg, Dingemanse et al. 2002; Quinn and Cresswell 2005), reptiles (e.g, Brodie and Russell 1999; Bajer et al. 2015; Horváth et al. 2017), mammals (eg, Schuster et al. 2017; Rorher and Ferkin 2020), fish (eg, Fuiman and Cowan 2003; Satterfield and Johnson 2020), and invertebrates (eg, Cordes et al. 2013; Kralj-Fišer and Schuett 2014). In addition to the overall consistency with studies of similar aspects of personality in other species, our study also supports the idea that the repeatability of a behavior can vary across populations of the same species (Dingemanse et al. 2002; Baker et al. 2018). Behavioral traits we measured exhibited differences in repeatability between the Palos Verdes and Catalina Island populations.

To understand why the level of consistent individual variation in behaviors may differ among populations, it is useful to consider the demographic consequences of individual variation in behaviors in the field. We found that behavioral traits of snails can be significantly associated with growth, survival, and biomass, and therefore fitness in the field. Moreover, there was spatial variation in patterns of selection, and we found that combinations of behavioral traits that lead to high fitness in 1 location may be neutral or even associated with low fitness in another location. For example, snails in the White Point population experienced strong and negative selection on exploration, whereas snails in the nearby population of Bailey's Reef experienced weakly positive selection on exploration. Similarly, snails with a combination of low exploration and low risk taking had relatively high fitness at Cherry Cove, but snails with the same combination of behaviors had low fitness at nearby Isthmus Reef. Such results are consistent with our first hypothesis of spatial variation in selection, which is in general believed to be an important force promoting variation in animal personality (Dingemanse and Réale 2005; Kight et al. 2013). Our results complement other recent studies showing spatial variation in selection on behavioral traits (Lapiedra et al. 2018; Mouchet et al. 2021; Brehm and Mortelliti 2024).

We had also hypothesized that patterns of selection would be qualitatively different between mainland regions, which were characterized by abundant, but slow-moving predators, and island regions which were characterized by scarcer, but fast-moving predators. There was only limited support for this hypothesis. Relationships between behavioral traits and growth were not very similar for nearby locations, suggesting that the local predator community had relatively little influence on selection via differential growth. Relationships between behavioral traits and survival had more similarities, and the biggest contrast between mainland and island regions was that selection on exploration was strongly stabilizing for the mainland populations, but relatively flat at the island populations. The mainland region had much higher densities of whelks and sea stars—predators that have low mobility and are dispersed regularly across the reef. At mainland locations, snails with the highest exploration scores had reduced survival rates, and our results are consistent with the idea that greater exploration can lead to a higher encounter rate with predators and thus a greater mortality risk (eg, Luttbeg and Schmitz 2000; Campos-Candela et al. 2019). However, reasons for snails with the lowest exploration scores also having lower survival are less clear. It may be that low exploration is associated with low energy reserves and thus lower ability to escape predators (eg, Schmitt 1981), despite fewer encounters overall. At the Island locations, there was little association between exploration and survival. At these locations whelks were absent and the primary predators were lobsters—a species that is scarce but highly mobile. At these locations, differences in exploration by snails would be unlikely to affect encounter rate since lobster are much more active and encounter rate is more likely to be driven by the behavior of lobsters, rather than snails (Withy-Allen and Hovel 2013).

Another idea that motivated this study was the hypothesis that traits such as exploration would be positively associated with growth but negatively associated with survival, thus leading to a combined fitness surface that is neutral, or even concave upward and indicative of disruptive selection. We saw little evidence to support this hypothesis. At Bailey's Reef, disruptive selection on exploration (via differential growth) was countered somewhat by stabilizing selection (via differential survival). However, patterns of selection via differential growth and selection via differential survival were either similar or reinforcing (eg, at White Point) or were weak and without meaningful similarities or differences between growth and survival selection (eg, Isthmus Reef, Cherry Cove). Although behaviorally mediated tradeoffs between growth and survival is a viable hypothesis for the maintenance of behavioral trait variation (Stamps 2007; Réale et al. 2010), we found that for wavy turban snails, it was more likely that a single fitness component could generate disruptive or stabilizing selection. For example, at Bailey's Reef, the most exploratory snails grew faster, possibly because of greater access to food. Wavy turban snails feed on algae, including detached pieces of giant kelp (Mazariegos-Villarreal et al. 2017). Their food is patchily distributed, and greater access to kelp increases growth rates (Martone and Micheli 2012). The least exploratory snails also exhibited high growth, possibly because less exploration and movement results in less energy expenditure and more energy available for growth, despite lower energy intake. For context, some snails in this study were recaptured only centimeters from where they were released while others were recaptured 10's of meters away. Although divers did not mark the locations of each individual's release and capture points, and it is not clear whether snails moved away but returned to their release point, these observations suggest the possibility of large differences in expenditure among individuals. These results are consistent with theory (Campos-Candela et al. 2019) and encourage further study of how personality variation is associated with energy budgets.

Understanding how selection acts through differences in biomass production provided a thorough measure of relative fitness, since biomass combines the effects of both growth and survival and reproduction is tightly linked to body size for our study species (Martone and Micheli 2012). Calculating biomass production also allowed a closer look at the relative importance of growth and survival. We note that in general, the shape of the biomass fitness surfaces tended to resemble the growth fitness surfaces more closely. The reason for this was that behavioral traits were associated with stronger differences in the *relative* values of growth and because growth had an indirect effect on survival. Survival increased with body size in all locations (though the shapes of the curves were somewhat different; see Table 3) and faster growth had an indirect and positive effect on survival. Figure 4 and accompanying analyses illustrate the direct effect of behaviors on survival whereas the calculation of relative biomass production includes the indirect effect of growth on cumulative survivorship.

Although the focus of this study was on field measurements of phenotypic selection on behavior at several locations, the results have implications for the long-term maintenance of variation in personality. Spatial variation in the direction and form of selection may be an important mechanism promoting variation in behavioral traits. Just as stabilizing selection can cause allele fixation and thus erode genetic variance, disruptive selection may promote variability (Rueffler et al. 2006; Pélabon et al. 2010). In this study, the degree of stabilizing vs. disruptive selection varied across traits and across locations. For example, there was significant stabilizing selection at White Point and significant disruptive selection at Bailey's Reef (see Fig. 5 and Table 4). In addition, there were differences in directional selection. Over time, such differences in the selection surfaces may promote genetic variance in behavioral traits as larvae are likely exchanged between these nearby locations (Zhang and Hill 2005). However, our comparison of the biomass fitness surfaces (pooled within regions) and the level of individual (co)variation in behaviors in both mainland and island regions suggests that understanding the net effects of selection in a region may require the sampling of additional populations.

The results of this study did not support the final hypothesis that the degree of repeatable variation in behavioral traits would be related to the strength of nonlinear selection. We found no evidence that degree of among-individual (co)variation in behavioral traits (see Fig. 2) was correlated with our estimates of the strength of nonlinear selection on those behaviors within regional populations (see Table 4). Results from the repeatability studies suggest a strong association between exploration and risk taking, a pattern that could arise from positive correlational selection (eg, Cheverud 1984; Sinervo and Svensson 2002; Chantepie and Chevin 2020). Correlational selection was strong and positive for both island locations, but not for the mainland locations. Similarly, the significantly greater among-individual component of variation in exploration at Santa Catalina Island could have resulted from weaker stabilizing selection on this trait in the island locations. Instead, the opposite pattern was observed (see pooled results in Table 4). However, a salient result of our study is that patterns of selection on behavioral traits can vary substantially between nearby populations. In light of these results, it may be that 2 study sites per region is simply not enough to accurately characterize the net pattern of selection experienced by regional populations. Given the likely distances of larval dispersal for our study species (del Próo et al. 2003), it is likely that behavioral trait variation reflects selection history at a spatial scale of kilometers to tens of kilometers. There were relatively few trait-population combinations to compare because of the labor-intensive and large-scale nature of the study, but results from our field studies suggest that substantial variation in selection can occur over these scales.

Overall, our study shows how selection on personality traits can vary at both local and regional scales and we encourage more studies of the mechanisms linking behavioral traits to relative fitness in natural populations. Such studies are challenging and relatively rare, but the continuation and expansion of this research will be critically important for explaining why personality variation exists within animal populations and how such variation is likely to be maintained in the future (see reviews by Moiron et al. 2020; Laskowski et al. 2022).

## Supplementary Material

araf146_Supplementary_Data

## Data Availability

Analyses reported in this article can be reproduced using the data provided by (Nguyen and Johnson 2025).
