# Supplementary material for: Personality variation in a marine snail and heterogeneous selection in natural populations
Source: Behav Ecol. 2025 Dec 19;37(1):araf146. doi: 10.1093/beheco/araf146 (PMC12797315; doi:10.1093/beheco/araf146)
Supplement: araf146_Supplementary_Data [file araf146_supplementary_data.docx]

**Personality variation in a marine snail and heterogeneous selection in natural populations**

**Supporting Online Material**

**Appendix S1: Predator and snail densities**

*Snail and Predator Abundances*

At the beginning of the mark-recapture studies, we surveyed the densities of snails and predatory species. Predators were counted on randomly placed transects that measured 30m long by 2m wide (n=6 per location). The four field sites differed in their densities of wavy turban snails. One mainland site (Bailey’s Reef: 1.583 individuals/m^2^ ± 0.1 SE) and one island site (Cherry Cove: 1.764 ± 0.317) had similarly high densities, whereas the other two sites had comparatively lower densities of wavy turban snails (Isthmus Reef: 0.783 ± 0.168, White Point: 0.931 ± 0.178 Figure S1A). In addition, the composition of the predator community differed strongly between mainland and island sites. Kellet’s whelks (*Kelletia kelletii)* are an abundant, but slow-moving predator and were observed only at the mainland sites (Figure S1B). Sea stars were found mostly at the mainland sites and were very rare at Catalina Island (Figure S1C). Spiny lobsters (*Panulirus interruptus*) were more abundant at the island sites, and although their overall abundance was low compared to whelks, lobsters are fast-moving and have a high per-capita impact on wavy turban snails. Octopus were rare and slightly more abundant at the mainland sites. Throughout the entire study, no *Kelletia* were observed at the island sites, but *Pisaster*, *Orthasterias*, *Panulirus* and *Octopus* were observed at all locations, even if not encountered within the sample of survey transects.

| 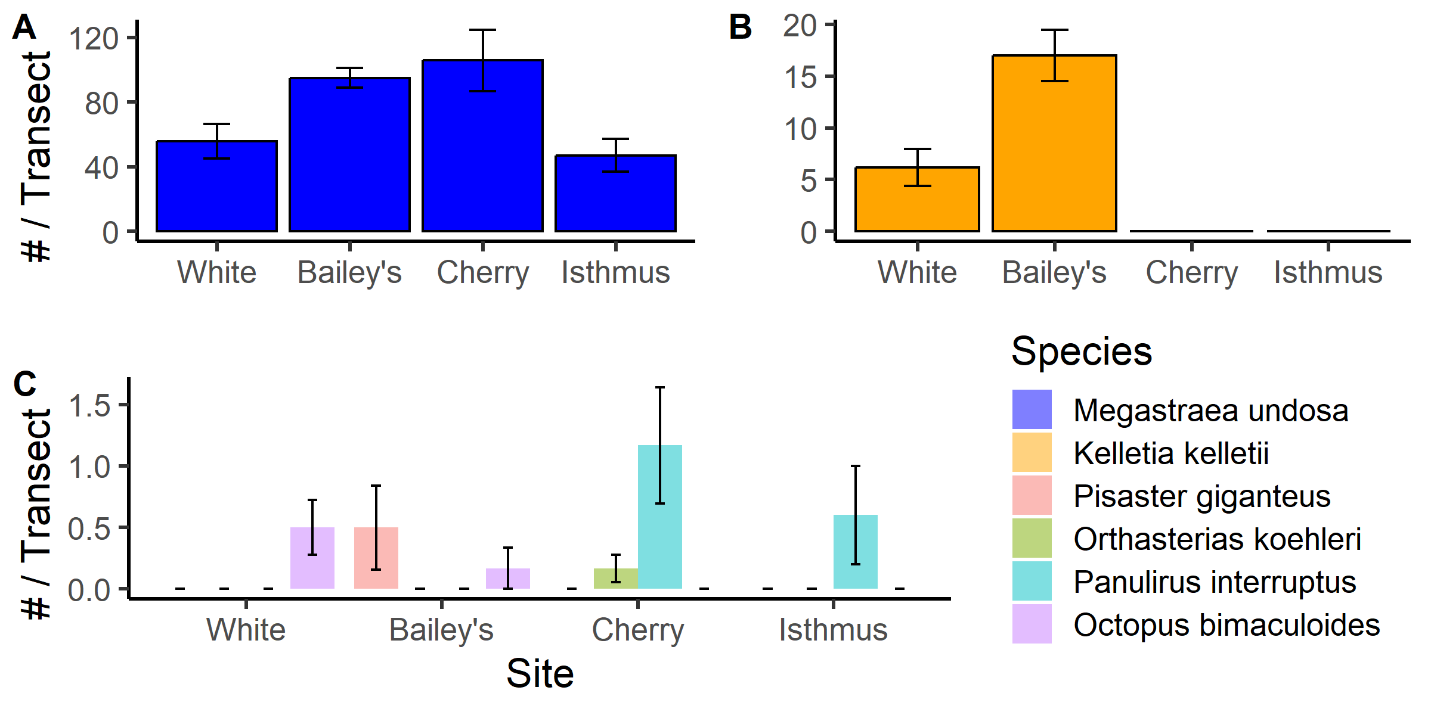 |
| --- |
| Figure S1. Average number of individuals (±1 SE) of each species per 60m^2^ (30 x 2 meter transects). A: Wavy turban snails, B: Kellet’s whelks, C: Other, less common predators. White point and Bailey’s Reef are mainland locations whereas Cherry Cove and Isthmus Reef are island locations. |

**Appendix S2: Estimating Tag Loss and Overgrowth**

**Tag loss:**

It is useful to estimate rates of tag failure, as tag failure may seriously bias recapture and survival parameter estimates (Malcolm-White et al. 2020; Mellado et al. 2022). Since all snails in this study were double tagged initially, we used observations of the number of tags on a snail upon recapture to calculate the rate of tag loss at each site. Following the procedure described by McCann and Johnson (2021), the probability of a double-tagged snail being recaptured and having both tags still attached is *r*/(2-r), where *r* is the probability of tag retention. For each group of snails recaptured, the number of snails retaining both tags divided by the total number of snails recaptured (i.e., including those that returned with only a single tag) provides an estimate of *r*/(2-r). Moreover, the observed number of double-tagged snails returning in a sample of *n* snails can be treated as a binomially distributed random variable. This allows the statistical likelihood to be calculated for any hypothesized value of *r*. In field studies, the tag retention probability (*r*) is unlikely to be static. Rather, tag retention probability is likely to decline over time, as glue may weaken over time, and more time at liberty means more opportunity for tags to rub off. In this study, the probability of a tag being retained was modeled as a negative exponential function of time (i.e., *r* = exp(-λt)). Data for all recaptures at a site were pooled, and we used maximum likelihood estimation to find the value of λ that was most likely to have produced the observed data. The accompanying 95% confidence limits were calculated by finding the lower and upper values of λ that reduced the log-likelihood by 1.92 units (Meeker & Escobar 1995).

*Results:* The proportion of double tagged snails that were recaptured with both tags intact decreased over time, but at a gradual rate (Figure S2). These results suggest that the rates of tag loss were modest. Values of λ (exponential rate of decline in the probability of tag retention)) were estimated for each site to be: White = 0.0006 d^-1^ (95% CI: 0.0004, 0.0009), Bailey’s = 0.0006 d^-1^ (95% CI: 0.0003, 0.0011), Cherry = 0.0005 d^-1^ (95% CI: 0.0003, 0.0010), Isthmus = 0.0015 d^-1^ (95% CI: 0.0009, 0.0024). Three of the sites showed similar rates of tag loss, while Isthmus Reef was the only site to have a significantly higher rate of tag loss (~24% loss over a 100-day period compared to an average of ~10% for the other three sites).


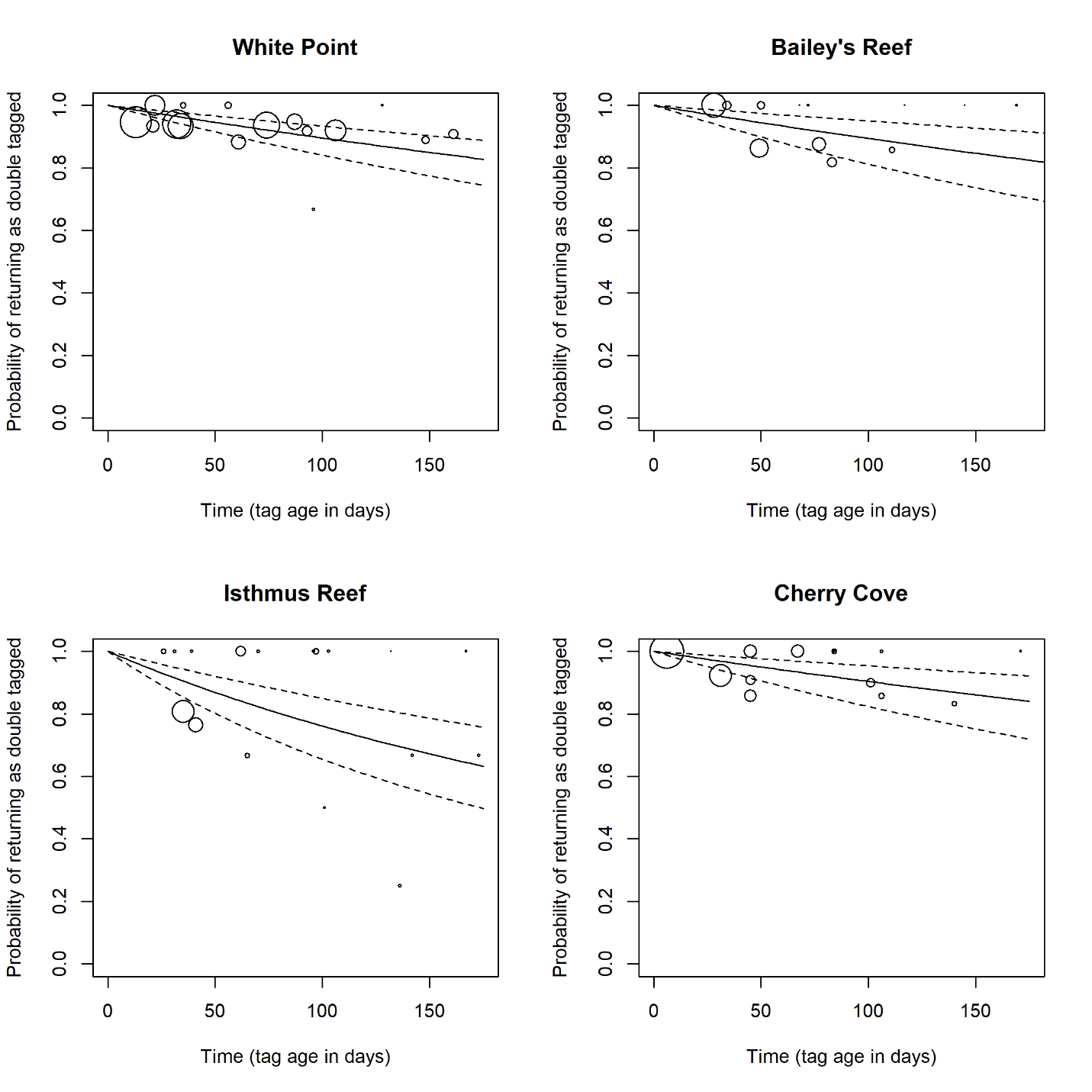


Figure S2. Tag loss. Panels display the probability that double tagged snails released in the field were captured with both tags present at a later point in time. Each data point represents a sample of snails and the size of the circle represents the sample size (smallest circle: n = 1, largest circle: n = 40). The probability of returning as double tagged for a particular tag age was calculated as: # of snails recovered with both tags / (# of snails recovered with both tags + # of snails recovered with a single tag). Curves are based on the estimated rate of time-dependent tag loss in an exponential model. Dashed lines represent 95% confidence bands.

**Tag overgrowth:**

Another form of tag failure that needed to be considered was the tag being overgrown with encrusting organisms and thus undetectable in the field. Tag overgrowth data was measured visually when snails were recaptured, and scored on a scale of 0 (clean) to 1 (completely overgrown). Because each snail had 2 tags, we used the average overgrowth across both tags as our unit of replication. We used a log linear regression of tag overgrowth on tag age to estimate the rate of tag overgrowth. No intercept term was included because all tags were clean upon deployment (i.e., when time = 0). This analysis thus describes the probability of tag overgrowth as an exponential function of time spent at liberty in the field: Pr[overgrowth] = exp(*Ct*), where *t* is time and *C* is the exponential rate of tag overgrowth. For display, tag visibility was expressed as 1 – overgrowth score and average tag overgrowth was expressed as 1 - exp(*Ct*). We calculated the rate of tag overgrowth separately for each site, as the rate of encrusting growth likely depends on nutrients and the number of propagules in the local area (Wernberg et al. 2010). Additionally, for the main mark-recapture analysis, we needed to quantify the probability that a snail of a certain tag age would still be visible in the field (i.e., not have both tags overgrown). The growth of encrusting organisms on the two tags of a snail are not independent processes, so we fitted the same type of model to the *minimum* tag overgrowth measurement between the two tags instead of the average overgrowth. The estimated coefficient of the log-linear model (α) thus allowed us to express the probability that a tagged snail was identifiable (i.e., at least one tag was not overgrown) as function of time: Pr[visible] = 1- exp(*αt*).

*Results:* The rate of tag overgrowth due to encrusting organisms colonizing tag surfaces was appreciable in this study. The average probability of a tag being visible declined steadily with time and the rates of decline in tag visibility appeared to differ by site. An exponential model of decline with time (visibility = 1- exp(*C*t)) fit the data well (Figure S3, blue curve), and the rates of decline (*C* values) for the various study populations were as follows: White Point = 0.0075 d^-1^ (95% CI: 0.006, 0.009), Bailey’s Reef = 0.0046 d^-1^ (95% CI: 0.004, 0.005), Cherry Cove = 0.0055 d^-1^ (95% CI: 0.004, 0.007), Isthmus Reef = 0.008 d^-1^ (95% CI: 0.006, 0.010). To put this into perspective, after 100 days out in the field, the average tag at Isthmus Reef would be 55% overgrown with algae, while the average tag at Bailey’s Reef would only be 37% overgrown (Figure S3). The visibility of at least one tag on a double tagged snail with time (*α* values) were as follows: White Point = 0.0059 d^-1^ (95% CI: 0.004, 0.007), Bailey’s Reef = 0.0028 d^-1^ (95% CI: 0.002, 0.003), Cherry Cove = 0.0043 d^-1^ (95% CI: 0.003, 0.005), Isthmus Reef = 0.0072 d^-1^ (95% CI: 0.005, 0.009) (Figure S3, red curve).


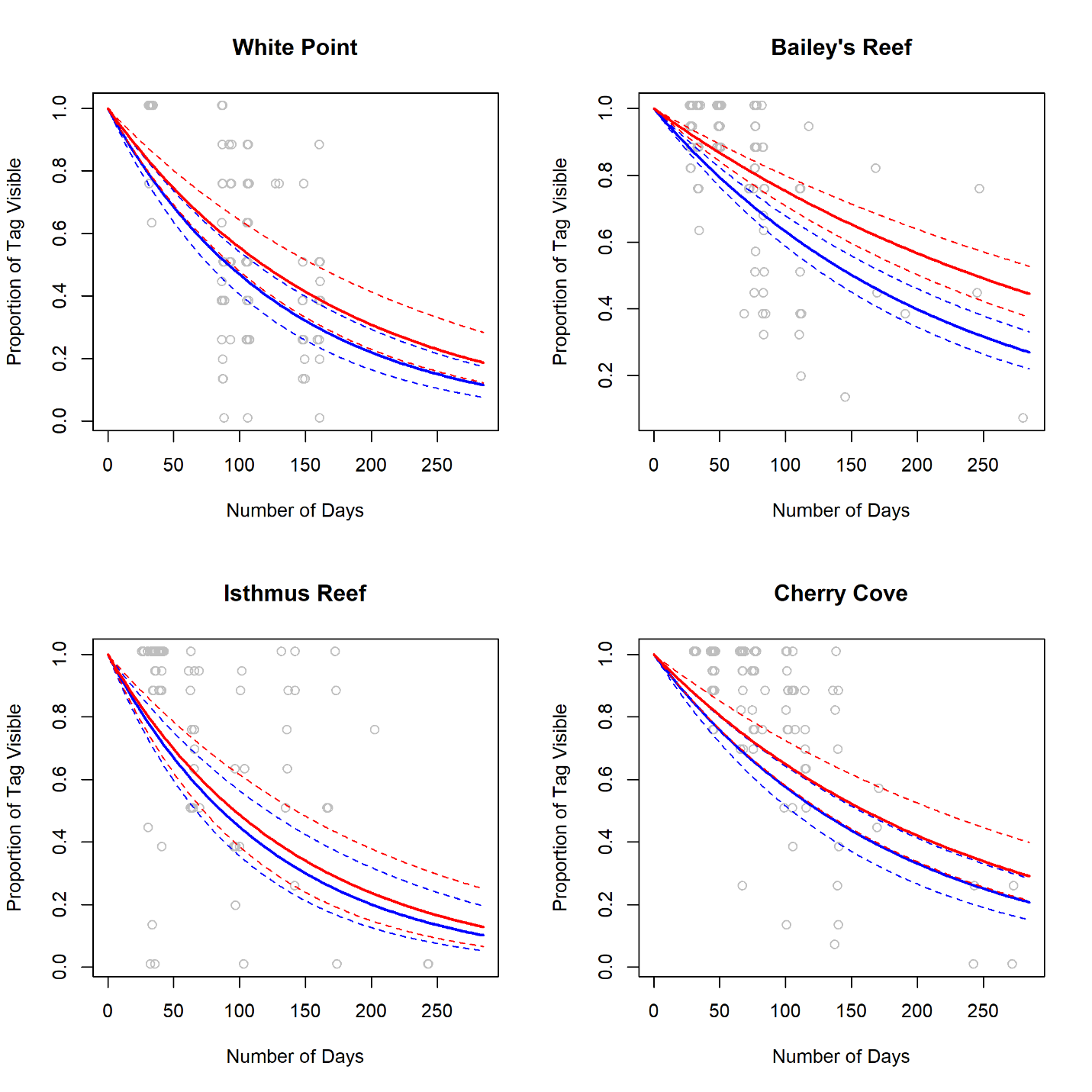


Figure S3. Tag overgrowth. Plots show the decline of tag visibility over time as tags on snails in the field became overgrown. Points represent the fraction of the tag that was visible for each individual. Individuals began the study with two tags and each data point represents the average overgrowth of an individual’s tags. The blue curve represents the average probability of a single tag being visible as an exponential function of tag age, estimated from algae growth on the average tag. The red curve represents the probability that at least 1 tag out of 2 on an individual is still visible with time, estimated from the minimum algae growth between two tags on an individual. Individuals with both tags overgrown were considered no longer recapturable. Dashed lines represent associated 95% confidence bands.

These results show that tag loss and tag overgrowth need to be accounted for in order to get accurate estimates of recapture and survival in a field mark-recapture study. The importance of factoring in tag loss in mark recapture models is already well understood and is a common practice in many studies (e.g., Björnsson et al. 2011; Cowen & Schwarz 2006; Mellado et al. 2022). However, our study shows that tag overgrowth may be of greater importance and can significantly alter recapture probabilities as well. For example, after tagged individuals were deployed for 100 days at Bailey’s Reef, 37% of the tags were completely overgrown, whereas only 10% of the tags had fallen off. We suspect that in aquatic systems, rates of tag overgrowth can be especially high and accounting for tag overgrowth will be essential for accurate inference from mark recapture studies. Studies should also consider that rates of tag loss and tag overgrowth can vary by location, as seen in this study. Local environmental conditions such as light availability, number of algae propagules, and water flow all likely play a role in influencing site-specific rates of tag loss and tag overgrowth.

The probability of a snail being identifiable was incorporated into the mark-recapture analysis as follows. For the unobservable states, transition probabilities to these states were calculated from our separate analyses of tag loss and overgrowth (see results below) and fixed within the mark-recapture analysis. Transition probability from state 1 to state L (a single tag snail losing its tag) within a time interval was fixed using the equation 1 - exp(-*λt*), where *t* represents the tag age and *λ* was estimated from our analysis of the return of double tagged snails (see results below). Transition probability from state 2 to state L (a double tagged snail losing both tags) within a time interval was fixed to be (1 - exp(-*λt*))^2^. Transition probability from state 1 to state G was fixed to be exp(*Ct*), where *C* reflects the average rate of overgrowth for a single tag. Transition probability from state 2 to state G (a double tag snail having both tags be overgrown) within a time interval was fixed to be exp(*αt*), with α reflecting the rate at which both of two tags on the same snail were overgrown. Transition probabilities describing the gain of a tag or the undoing of overgrowth were fixed at zero.

**Supplemental Text S2: Comparing fitness surfaces between locations**

The nature of our study and the structure of our data precluded conventional tests of the null hypothesis that the selection surfaces via differential survival and via differential biomass production were the same for different locations. In particular, the encounter history dates were not the same for the study locations, and the high rate of tag overgrowth and loss (relative to the lifespan of the animal) necessitated the use a multistate model to measure survival accurately. We used the RMark program (Laake 2013) for fast, likelihood-based estimation of survival parameters all within the context of a multi-state model to account for tag loss and overgrowth. However, to our knowledge there was no straightforward way in which we could have pooled the data and used the RMark package to compare the fit of a model with a single selection surface to the fit of location-specific models with additional parameters, while also accounting for location-specific rates of tag loss and overgrowth and differences in the timing of censuses.

Instead, we used randomization tests to evaluate the statistical evidence that selection surfaces differed between locations. The essence of these tests was to calculate the probability of obtaining estimates of the fitness surface as different as we observed, if they in fact came from the same underlying surface, but were estimated with uncertainty. In cases with measurement uncertainty, one would not expect two estimates of the fitness surfaces to be *exactly* the same. Rather, one would expect most samples to mismatch to a small degree. Given the uncertainty that results from sampling variation, one can envision a distribution of mismatch values that are consistent with the idea of a single fitness surface. Our randomization test was designed to simulate this process.

For each pairwise comparison of locations, our null model was a common fitness surface that was derived by averaging the quadratic regression coefficients for the two locations (see Tables 3 and 2 of the main text), and averaging the associated variance-covariance matrices to characterize the expected degree of sampling variation for this null model. For each of 200 iterations, we sampled the null model twice to create two, randomly-drawn functions with uncertainty representative of what we observed in our field study. This procedure simulated the process of sampling two fitness surfaces (e.g., from two different locations) if the true fitness surface was actually the same. We then summarized the mismatch of the functions by calculating the square of the difference in relative fitness values predicted by the two functions at each trait combination, and then integrating across all possible trait combinations within the observed range. Expressed in symbolic notation, mismatch was measured as

$\iint\left( \hat{w_{1}\left( x,y \right)}-\hat{w_{2}\left( x,y \right)} \right)^{2}dxdy$ (S1)

Where *w*_1_ and *w*_2_ are resampled, relative fitness functions, and *x* and *y* are the trait values (standardized exploration and boldness scores in this study). The parameters of the relative fitness functions were drawn from a multivariate normal distribution with means defined by the vector of averaged quadratic regression coefficients and a variance-covariance matrix that was defined by the average of the estimated matrices from each location. For each of the 200 iterations, we applied expression S1 to obtain a measure of mismatch for two resamples of the null model. Two hundred iterations was a large enough sample to provide a thorough description of the expected distribution of mismatch values under the null hypothesis, but small enough to make the computations manageable on a desktop computer.

To compare our field results to the null expectations, we applied expression S1 to the fitness functions estimated by our main survival analyses (see Tables 2 and 3 of the main text) and then calculated the proportion of the expected mismatch values under the null model that were as or more extreme than the observed mismatch. This provided a P-value to test the null hypothesis that the underlying fitness surface was actually the same for the two locations.

**Appendix S3: Full summary of mark-recapture models**

| Table S1. Full summary of mark-recapture models for each of our study populations. Terms beginning with the letter S are component coefficients of the survival model, which depended on behavioral trait scores. Terms beginning with the letter p relate to recapture probabilities, which were estimated as a baseline value (intercept) plus single terms representing a component deviation for each census. Terms beginning with Psi are the mean probabilities of a snail losing a single tag and transitioning from the 2-tag state to the 1-tag state. Coefficients describe the logit of each of the probabilities (survival, recapture, and tag loss) and are expressed for the average time interval for each population (White Point =30.5 days, Bailey’s Reef = 48 days, Isthmus Reef = 29.65 days, Cherry Cove = 39 days) |
| --- |
| **** |

| **Table S2.** Mark recapture summaries with uncorrected coefficients relating measures of behavior (Best Linear Unbiased Predictions from the repeatability analysis) to survival. Abbreviations are as in Table S1. Coefficients describe the logit of each of the probabilities (survival, recapture, and tag loss) and are expressed for the average time interval for each population (White Point =30.5 days, Bailey’s Reef = 48 days, Isthmus Reef = 29.65 days, Cherry Cove = 39 days) |
| --- |
| **** |

| Table S3. Uncorrected coefficients relating measures of behavior (Best Linear Unbiased Predictions from the repeatability analysis) to growth. |
| --- |
| **** |

**REFERENCES**

Björnsson, Björn, Hjalti Karlsson, Vilhjálmur Thorsteinsson, and Jón Solmundsson. 2011. “Should All Fish in Mark - Recapture Experiments Be Double-Tagged? Lessons Learned from Tagging Coastal Cod (*Gadus Morhua*).” *ICES Journal of Marine Science* 68 (3). https://doi.org/10.1093/icesjms/fsq187.

Cowen, Laura, and Carl J. Schwarz. 2006. “The Jolly-Seber Model with Tag Loss.” *Biometrics* 62 (3). https://doi.org/10.1111/j.1541-0420.2006.00523.x.

Laake, J L. 2013. “RMark: An R Interface for Analysis of Capture-Recapture Data with MARK.” *AFSC Processed Rep. 2013-01*.

Malcolm-White, Emily, Clive R. McMahon, and Laura L.E. Cowen. 2020. “Complete Tag Loss in Capture–Recapture Studies Affects Abundance Estimates: An Elephant Seal Case Study.” *Ecology and Evolution* 10 (5). <https://doi.org/10.1002/ece3.6052>.

McCann, BN, and DW Johnson. 2021. “Estimating Growth, Size-Dependent Mortality, and Tag Loss in a Mark-Recapture Study: Demography of Wavy Turban Snails in Southern California, USA.” Marine Ecology Progress Series 659 (February). https://doi.org/10.3354/meps13604.

Meeker, William Q., and Luis A. Escobar. 1995. “Teaching about Approximate Confidence Regions Based on Maximum Likelihood Estimation.” *American Statistician* 49 (1). https://doi.org/10.1080/00031305.1995.10476112.

Mellado, Breno, Lucas De Oliveira Carneiro, Marcelo Rodrigues Nogueira, and Leandro Rabello Monteiro. 2022. “The Impacts of Marking on Bats: Mark-Recapture Models for Assessing Injury Rates and Tag Loss.” *Journal of Mammalogy* 103 (1). https://doi.org/10.1093/jmammal/gyab153.

Wernberg, Thomas, Fernando Tuya, Mads S. Thomsen, and Gary A. Kendrick. 2010. “Turban Snails as Habitat for Foliose Algae: Contrasting Geographical Patterns in Species Richness.” *Marine and Freshwater Research* 61 (11). https://doi.org/10.1071/MF09184.
